# Deoxyribonuclease is prognostic in patients undergoing transcatheter aortic valve replacement

**DOI:** 10.1111/eci.13595

**Published:** 2021-06-08

**Authors:** Andreas Mangold, Anna S. Ondracek, Thomas M. Hofbauer, Tyler Artner, Johanna Nechvile, Noel G. Panagiotides, Moritz Mirna², Matthias Hammerer, Dzeneta Fejzic², Uta Hoppe², Bernhard Wernly², Alexander Lauten, Brunilda Alushi, Marcus Franz, Paul C. Schulze, Evelyne Wohlschläger‐Krenn, Irene M. Lang, Michael Lichtenauer²

**Affiliations:** ^1^ Department of Internal Medicine II Division of Cardiology Medical University of Vienna Vienna Austria; ^2^ Department of Internal Medicine II Division of Cardiology Paracelsus Medical University of Salzburg Salzburg Austria; ^3^ Department of Cardiology Charité‐Universitätsmedizin Berlin Berlin Germany; ^4^ Department of Internal Medicine I, Division of Cardiology Pneumology, and Intensive Medical Care Friedrich‐Schiller‐University Jena Germany; ^5^ Center of Prevention and Health Sanatorium Hera Vienna Austria

**Keywords:** aortic valve stenosis, deoxyribonuclease, extracellular DNA, transcatheter aortic valve replacement

## Abstract

Degenerative aortic valve stenosis is an inflammatory process that resembles atherosclerosis. Neutrophils release their DNA upon activation and form neutrophil extracellular traps (NETs), which are present on degenerated aortic valves. NETs correlate with pressure gradients in severe aortic stenosis. Transcatheter aortic valve replacement (TAVR) is an established treatment option for aortic valve stenosis. Bioprosthetic valve deterioration promoted by inflammatory, fibrotic and thrombotic processes limits outcome. Deoxyribonuclease is a natural counter mechanism to degrade DNA in circulation. In the present observational study, we investigated plasma levels of double‐stranded DNA, deoxyribonuclease activity and outcome after TAVR.

345 consecutive patients undergoing TAVR and 100 healthy reference controls were studied. Double‐stranded DNA was measured by fluorescence assays in plasma obtained at baseline and after TAVR. Deoxyribonuclease activity was measured at baseline using single radial enzyme diffusion assays. Follow‐up was performed at 12 months, and mean aortic pressure gradient and survival were evaluated. Receiver operating characteristic, Kaplan‐Meier curves and Cox regression models were calculated.

Baseline double‐stranded DNA in plasma was significantly higher compared to healthy controls, was increased at 3 and 7 days after TAVR, and declined thereafter. Baseline deoxyribonuclease activity was decreased compared to healthy controls. Interestingly, low deoxyribonuclease activity correlated with higher C‐reactive protein and higher mean transaortic gradient after 12 months. Finally, deoxyribonuclease activity was a strong independent predictor of outcome 12 months after TAVR.

Deoxyribonuclease activity is a potential biomarker for risk stratification after TAVR. Pathomechanisms of bioprosthetic valve deterioration involving extracellular DNA and deoxyribonuclease merit investigation.

## 1 BACKGROUND

Aortic valve stenosis (AS) is the most frequent valve disease with growing prevalence in a population with increasing life expectancy. Without valve replacement, severe AS results in poor outcome.^1^ Transcatheter aortic valve replacement (TAVR) is an established treatment option for aortic stenosis patients with high and intermediate surgical risk^2^ and may even be considered for low‐risk patients.^3^ Results after TAVR are excellent.^2^ However, bioprosthetic valve deterioration is reported in around 40% of patients 20 years after surgery.^4^ The pathomechanism of bioprosthetic valve degeneration is not well understood, but includes thrombosis, pannus and accelerated calcification.^5^ Specific findings like hypoattenuating leaflet thickening might be an early sign of prosthesis degeneration.^6^ One important process may be a low‐grade inflammatory reaction to the biomaterial, involving neutrophils and macrophages.^7^ Neutrophils are able to undergo NETosis, in which the nucleus content is released into the extracellular space. These neutrophil extracellular traps (NETs) mainly consist of double‐stranded deoxyribonucleic acid (dsDNA) and granule proteins and are highly proinflammatory, cytotoxic, prothrombotic and profibrotic.^8^ Another important source of circulating dsDNA is destructed cells. Endogenous deoxyribonuclease (DNase) is present in plasma and is a natural counter mechanism to degrade DNA in circulation.^9^ In a murine model, it was reported that intact DNase function in circulation is vital.^10^ We have previously shown that coronary thrombus NET burden and DNase activity correlate with infarct size in ST‐elevation myocardial infarction patients^11^ and that circulating dsDNA and DNase predict survival in patients after cardiac arrest.^12^ High NET burden promotes fibrosis in mice and in patients.^13^ Double‐stranded DNA in plasma is a robust surrogate marker of NET burden in the circulation.^14^ Recently, it was shown that cusps of native high‐grade aortic stenosis contain neutrophils undergoing NETosis and that cuspal NET burden correlates with mean transaortic gradient.^15^ Biomarkers for prognosis of AS patients are interesting both for pre‐interventional evaluation and for postinterventional monitoring. In the present study, we measured dsDNA levels and DNase activity in AS patients undergoing TAVR and correlated these markers with survival after 12 months.

## 2 METHODS

### 2.1 Patients

A total of 345 patients suffering from symptomatic, severe AS and planned for TAVR were enrolled in this study between 2010 and 2018. 241 patients were recruited at the Department of Internal Medicine I, Division of Cardiology, Pneumology, and Intensive Medical Care, Friedrich‐Schiller‐University, Jena, Germany, and 104 patients were recruited at the Department of Internal Medicine II, Division of Cardiology, Paracelsus Medical University of Salzburg, Austria. From 79 patients, blood was obtained at baseline, after 7 days and after 1, 3 and 6 months; from 104 patients, blood was obtained at baseline, after 1 day and 3 days and after 3 months; and from 162 patients, blood was obtained at baseline.

The diagnosis of severe AS was defined according to the guidelines of the European Society of Cardiology. All patients underwent pre‐interventional examinations (electrocardiography, laboratory parameters, transthoracic and transoesophageal echocardiography and coronary angiography). Coronary artery disease was defined as proof of coronary atherosclerotic lesions in coronary angiography. Results were discussed in the interdisciplinary heart team. TAVR was chosen by the team for patients in whom the risk‐benefit ratio favoured a less invasive procedure.

TAVR was performed using Edwards Sapien, Medtronic Core Valve and JenaValve prostheses at the Jena centre and Medtronic Core Valve at the centre in Salzburg via transfemoral access in 84.8% and via transapical approach in 15.2% of all patients enrolled. In patients with transfemoral access, a 14‐21 French delivery sheath was placed into the femoral artery and a temporary transcutaneous pacemaker was placed. For transapical approach, an anterolateral mini‐thoracotomy was performed to access the apex of the left ventricle. After pericardiotomy, the apex was punctured by a standard access needle. A 12 French sheath was placed in the apex, rapid ventricular pacing initiated and valvuloplasty performed. The valves were placed with fluoroscopic guidance. For Medtronic CoreValve prostheses, no pacing was needed.

Healthy control probands (n = 100) were recruited in the Centre of Prevention and Health, Sanatorium Hera, Vienna, Austria. They are not age‐matched and were included in the study as a reference control cohort.

Blood samples were obtained by cubital venous blood draws using citrate‐anticoagulated tubes. After centrifugation at 200g for 10 minutes, plasma was frozen in aliquots at −80°C until the assays. Transportation was kept at a minimum in dry ice to maintain freezing conditions until the assays.

The study protocol was approved by the Ethics Committee of the Friedrich‐Schiller‐University, Jena, Germany (3237‐09/11), by the Ethics Committee of the County of Salzburg (415‐E/1969/5‐2016) and by the Ethics Committee of the Medical University of Vienna (1947/2014). The study was conducted according to the principles of the Declaration of Helsinki and Good Clinical Practice. Written informed consent was obtained from all patients before enrolment. Reporting of the study conforms to broad EQUATOR guidelines.^16^

### 2.2 Routine blood count and plasma analyses

Blood draws for routine laboratory testing including CRP and BNP were obtained at baseline from all patients and measured in the routine laboratory in the Paracelsus Medical University of Salzburg, Austria, and the University Hospital of Jena, Germany in the course of routine clinical care.

### 2.3 Double‐stranded DNA measurement

Double‐stranded DNA was measured as reported before.^17^ Sytox Green (Thermo Fisher) was added to plasma samples diluted 1:20 or standard (lambda DNA, Thermo Fisher) for 5 minutes, and fluorescence was measured using a Synergy H1 Hybrid microplate reader (excitation 480 nm, emission 520 nm, BioTek). Fluorescence intensities were normalized to the standard curve.

### 2.4 Deoxyribonuclease activity measurement

Total DNase activity was determined as reported before.^12^ Salmon testes DNA was used at a concentration of 100 μg/ml in assay buffer containing positive divalent ions and DNA binding fluorescent dye (35 mM Tris‐HCl pH 7.8, 20 mM MgCl2, 2 mM CaCl2 and 2.5x SYBR Safe). The solution was heated to 50°C for 10 minutes prior to addition of an equal volume of 2% agarose. The mixture was poured into a plastic tray for solidification. 2 μl plasma was loaded into wells of 1 mm diameter and incubated for 24 hours at 37°C in a humid chamber. Remaining fluorescence of gels was recorded using a fluorescence scanner after 6, 10 and 20 hours.

### 2.5 Statistics

Distribution of data was assessed by histograms complemented by Kolmogorov‐Smirnov test and Shapiro‐Wilk test. Data not following a normal distribution are given as median and interquartile range (IQR) and were analysed using Mann‐Whitney U test, Wilcoxon signed‐rank test and Spearman rank correlation (r_s_). Normally distributed data are given as mean ± standard deviation (SD) and were tested using paired or unpaired Student's *t*‐test and Pearson correlation. Multiple comparisons were corrected with the Bonferroni‐Holm method. Time course measurements were evaluated using analysis of variance repeated measurements with Bonferroni *post hoc* evaluation. More than two groups were compared by analysis of variance with Dunnett‐T3 *post hoc* evaluation. For comparison of plasma levels, only paired data points were used in the tests. A receiver operating characteristic curve was applied to determine the optimal cut‐off value calculated by the Youden index. A Kaplan‐Meier curve was plotted, and log‐rank test was calculated. Univariate and multivariate Cox regression models were calculated to identify significant predictors of outcome. Data are presented as box plots, and whiskers indicate 5% and 95% percentile. Figures were generated using GraphPad Prism 8.0 and Adobe Illustrator CS6. A p‐value below 0.05 was considered significant. Statistical testing was performed using IBM SPSS Statistics 22.0 for Windows.

## 3 RESULTS

### 3.1 Patients

345 patients were enrolled in this study. Median age was 82 years (IQR 78‐85), and 182 patients were female (53.9%). Median body mass index (BMI) was 27.1 kg/m² (IQR 24.1‐30.4). 22% of patients were not studied at 12 months. 68 patients deceased within 12 months after TAVR.

Baseline serum level of C‐reactive protein (CRP) was 20.9 nmol/l (IQR 0.0‐89.52), concentration of brain natriuretic peptide (BNP) was 96.7 pmol/l (IQR 37.3‐282.3), and estimated glomerular filtration rate was 59.4 ml/min/1.73 m² (IQR 44.6‐70.0, all medians). Baseline left ventricular ejection fraction (EF) was 60.0% (IQR 50.0‐67.0).

Mean pressure gradient before TAVR was 46.4 ± 15.4 mmHg (n = 322), after TAVR, it was 9.5 ± 5.2 mmHg (n = 184), and 12 months after TAVR, it was 9.3 ± 6.6 mmHg (n = 90).

Mild periprocedural vascular complications occurred in 21.2% (n = 73), and severe vascular complications occurred in 6.7% (n = 23). Stroke within 30 days occurred in 3.2% (n = 11). After TAVR, mild paravalvular leak occurred in 43.2% (n = 149), moderate paravalvular leak occurred in 12.5% (n = 43), and severe paravalvular leak occurred in 0.6% (n = 2). Aortic regurgitation after 12 months was mild in 64.8% (n = 59) and moderate or severe in 5.5% (n = 5).

Median age of healthy reference controls (n = 100) was 65 years (IQR 60‐71), and 49 probands were female. BMI was 25.4 kg/m² (IQR 23.2‐28.9), CRP was 10.48 nmol/l (IQR 4.76‐23.81), and estimated glomerular filtration rate was 77.1 ml/min/1.73 m² (IQR 67.3‐89.5, all medians). BNP of healthy reference controls was 6.4 pmol/l (IQR 5.6‐7.1). Detailed parameters are given in Table 1.

**TABLE 1:** Patient demographics

	TAVR recipients (n = 345)		Healthy ref. controls (n = 100)	
	median	IQR	median	IQR
Age (y)	82	78‐85	65	60‐71
BMI (kg/m²)	27.1	24.1‐30.4	25.4	23.2‐28.9
Haemoglobin (mmol/l)	7.7	7.0‐8.5	9.0	8.6‐9.4
CRP (nmol/l)	20.9	0.0‐89.52	10.5	4.76‐23.81
Ejection fraction (%)	60.0	50.0‐67.0		
BNP (pmol/l)	96.7	37.3‐282.3	6.4	5.6‐7.1
Creatinine (µmol/l)	97.0	76.0‐124.0	80.4	71.6‐91.9
eGFR (ml/min/1.73 m²)	59.4	44.6‐67.0	77.1	67.3‐89.5
	**%**	**n**	**%**	**n**
Female gender	53.9	124	49	49
Diabetes mellitus	41.3	142	4	4
Arterial hypertension	90.7	312	46	46
Coronary artery disease	68.6	236	0	0
Peripheral artery disease	23.3	80	0	0
Stroke 12 mo FU)	5.5	19	0	0
MI (12 mo FU)	2.2	8	0	0
PM (12 mo FU)	26	94	0	0

Abbreviations: BMI, Body mass index; BNP, Brain natriuretic peptide; CRP, C‐Reactive protein; eGFR, Estimated glomerular filtration rate; healthy reference controls (healthy ref. controls); IQR, Interquartile range; MI, Myocardial infarction; PM, Pacemaker implantation; TAVR, Transcatheter aortic valve replacement; 12 mo FU (12 mo follow‐up).

### 3.2 Double‐stranded DNA is increased in transcatheter aortic valve replacement patients

**FIGURE 1:**
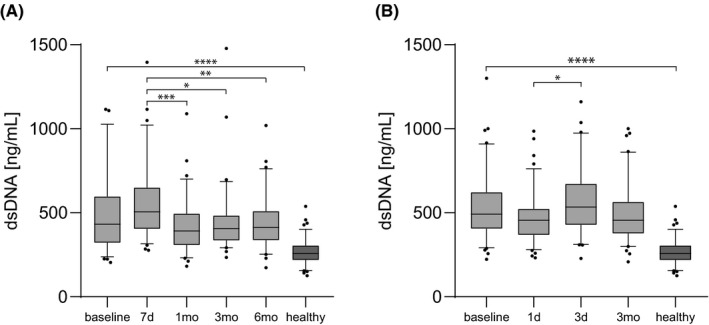
Double‐stranded DNA levels in transcatheter aortic valve replacement patients. Levels of dsDNA were measured in TAVR patients. A, n = 79: before TAVR (baseline), after 7 days (7d), 1 month (1mo), 3 months (3mo) and 6 months (6mo); B, n = 104: before TAVR (baseline), after 1 day (1d), 3 days (3d) and 3 months (3mo). Non–age‐matched healthy control probands served as a reference control cohort (healthy, n = 100). Analysis of variance repeated measurements with Bonferroni correction and Mann‐Whitney *U* test was applied. *P < .05, **P < .01, ***P < .001 and ****P < .0001. Double‐stranded DNA (dsDNA) and transcatheter aortic valve replacement (TAVR)

Double‐stranded DNA levels were measured in all patients at baseline and at different time points after TAVR (Figure 1). TAVR patients displayed higher baseline dsDNA levels than the healthy reference control group (TAVR median 476.3 ng/ml [IQR 373.7‐619.7] vs healthy median 256.6 ng/ml [IQR 217.2‐304.7]; Figure 1). Double‐stranded DNA was increased between 3 and 7 days after TAVR compared to baseline (Figure 1). Baseline dsDNA levels correlated significantly with BNP (baseline: r_s_=0.293, *P* < .0001, n = 183; after 3 months: r_s_=0.327, *P* < .001, n = 118). There was no correlation between baseline dsDNA levels and CRP, creatinine or EF (data not shown).

### 3.3 Deoxyribonuclease activity is low in transcatheter aortic valve replacement recipients and correlates with symptoms and mean pressure gradient after 12 months

**FIGURE 2:**
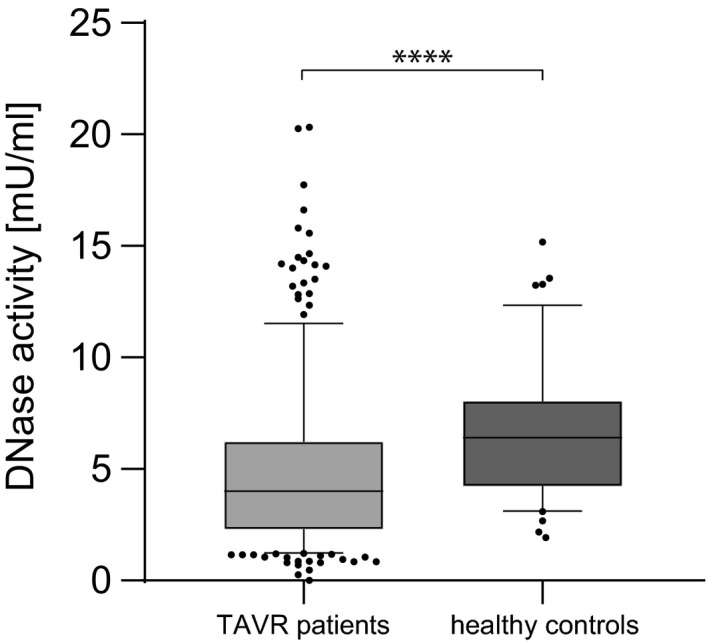
Deoxyribonuclease activity in transcatheter aortic valve replacement patients. DNase activity (n = 335, median 3.44 mU/ml [IQR 1.96‐6.09]) was significantly increased. Non‐age‐matched healthy control probands served as a reference control cohort (healthy, n = 100, median 6.4 mU/ml [IQR 4.25‐8.02]). Mann‐Whitney U test was applied. ****P < .0001. Deoxyribonuclease (DNase) and transcatheter aortic valve replacement (TAVR).

**FIGURE 3:**
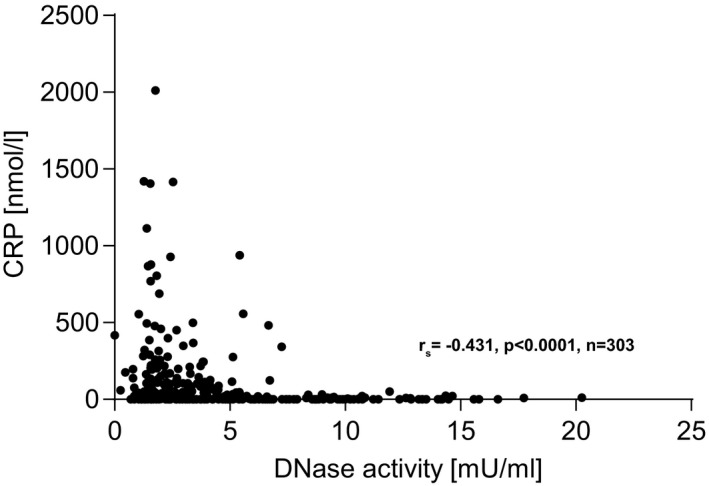
Correlation of deoxyribonuclease activity and C‐reactive protein in transcatheter aortic valve replacement patients. DNase activity correlated significantly with CRP determined prior to TAVR (n = 303). Spearman correlation was applied. C‐reactive protein (CRP), deoxyribonuclease (DNase) and transcatheter aortic valve replacement (TAVR)

**FIGURE 4:**
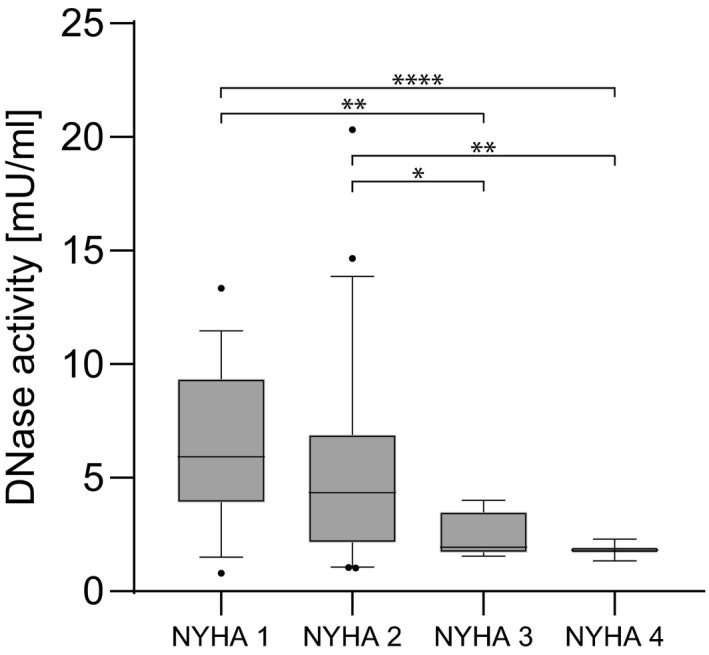
Deoxyribonuclease activity and NYHA class. TAVR patients (n = 86) at NYHA class 3 and 4 after 12 months had significantly lower DNase levels at baseline compared to patients at NYHA class 1 or 2. Analysis of variance and Dunnett‐T3 *post hoc* analysis were performed. *P < .05, **P < .01 and ****P < .0001. Deoxyribonuclease (DNase) and transcatheter aortic valve replacement (TAVR)

**FIGURE 5:**
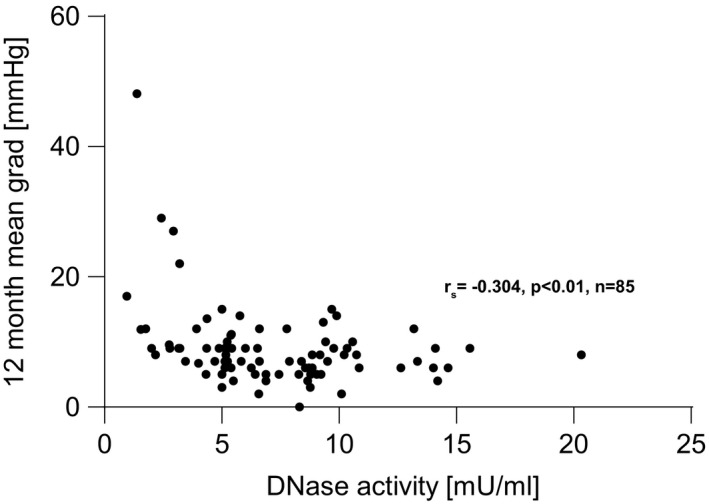
Deoxyribonuclease activity and mean aortic pressure gradient after 12 months. DNase activity correlated negatively with mean aortic pressure gradient (mean gradient) 12 months after TAVR (n = 85). Spearman correlation was applied. Deoxyribonuclease (DNase) and transcatheter aortic valve replacement (TAVR)

Total DNase activity was measured in baseline plasma levels from all patients and control probands. Compared to the healthy reference control cohort, DNase levels were low in TAVR recipients (TAVR median 3.44 mU/ml [IQR 1.96‐6.09] vs healthy median 6.4 mU/ml [IQR 4.25‐8.02]; Figure 2). In TAVR patients, a significant negative correlation between CRP and DNase activity (n = 303, r_s_=−0.431, *P* < .0001; Figure 3A) was observed. No correlations were found between DNase activity and glomerular filtration rate, EF or BNP (data not shown). DNase activity was significantly lower in NYHA class 3 (n = 7) and 4 (n = 2) compared to 1 (n = 34) and 2 (n = 43; Figure 4). Mean aortic pressure gradient after 12 months correlated with mortality (n = 147, r_s_=0.19, *P* < .05). DNase activity correlated negatively with mean aortic pressure gradient 12 months after TAVR (n = 85, r_s_=−0.304, *P* < .01; Figure 5A).

### 3.4 Deoxyribonuclease activity independently predicts outcome 12 months after transcatheter aortic valve replacement

**FIGURE 6:**
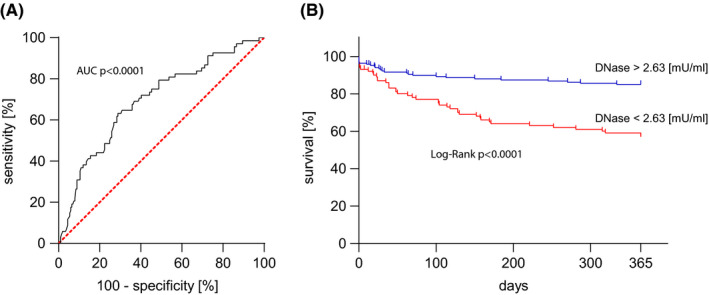
Deoxyribonuclease activity and survival after 12 months. A, Receiver operating characteristic curve. Baseline DNase activity predicts survival 12 months after TAVR (n = 269) with a sensitivity of 62% and a specificity of 71%, AUC *P* < .0001. B, Kaplan‐Meier plot. Baseline DNase activity independently predicts survival with a cut‐off of 2.63 mU/ml (n = 269, log‐rank test *P* < .0001). Area under the curve (AUC), deoxyribonuclease (DNase) and transcatheter aortic valve replacement (TAVR)

The power of DNase activity to predict outcome 12 months after TAVR was evaluated. In a receiver operating characteristic curve (area under the curve [AUC] 0.694, 95% confidence interval [CI] 0.621‐0.767, *P* < .0001), a cut‐off value of 2.63 mU/ml with a sensitivity of 62% and a specificity of 71% was identified by calculating the Youden index (Figure 6A). This binary variable (above or below 2.63 mU/ml) strongly predicted outcome of the patients in a univariate Cox regression model (hazard ratio [HR] 3.32, 95% CI 2.02‐5.43), *P* < .0001). 12 months after TAVR, 85.1% of patients with a DNase activity above 2.63 mU/ml were alive, in contrast to only 57.4% of patients with a DNase activity below 2.63 mU/ml (*P* < .0001; Figure 6B).

A multivariate Cox regression model was set up based on established clinical risk prediction scores (EuroSCORE II and STS score)^18^ and a published model, for which the strongest variables were evaluated to predict mortality after TAVR.^19^ Of 269 patients, 68 patients deceased within 12 months; therefore, the following 7 variables were chosen for the model: age, coronary artery disease, arterial hypertension, EF, creatinine, CRP and DNase activity. The metric variables were divided by the overall standard deviation to display the hazard ratio for change of 1 standard deviation of the variables. In this model, DNase activity proved to be a strong independent predictor for survival 12 months after TAVR (HR 3.11, 95% CI 1.56‐6.17, *P* < .001; Table 2). While CRP was significant in the univariate model (HR 1.24, 95% CI 1.10‐1.39, *P* < .0001), it lost significance in the multivariate model. EF also significantly predicted outcome (HR 0.71, 95% CI 0.52‐0.96, *P* < .05; Table 2) in the multivariate model.

**TABLE 2:** Effect of deoxyribonuclease activity on all‐cause mortality at 12 mo after transcatheter aortic valve replacement

Factor	Adjusted HR	95% CI	*P*‐value
Age	1.12	0.70‐1.80	.64
Coronary artery disease	1.10	0.53‐2.28	.81
Arterial hypertension	3.36	0.44‐25.41	.24
Creatinine	1.20	0.96‐1.50	.11
CRP	1.15	0.93‐1.42	.21
EF	0.71	0.52‐0.96	.028
DNase activity (cut‐off 2.63 mU/ml)	3.11	1.56‐6.17	.001

Note

Multivariable Cox regression was calculated to assess the influence of baseline DNase activity on all‐cause mortality after 12 mo (n = 269).

Abbreviations: CI, Confidence interval; CRP, C‐reactive protein; DNase, deoxyribonuclease; EF, Ejection fraction; HR, Hazard ratio; TAVR, Transcatheter aortic valve replacement.

## 4 DISCUSSION

In the present study, we observed increased circulating DNA and low DNase activity at baseline in patients with severe aortic stenosis. DNase activity emerged as a strong independent predictor of outcome 12 months after TAVR.

The main DNases present in plasma are DNase 1 and DNase 1L3 with distinct features.^20^ DNase 1L3 is released by macrophages and is discussed to be the predominant enzyme which degrades extracellular DNA in circulation.^21^ NET‐releasing neutrophils are one important source of extracellular DNA. In the past years, it was shown that NETs contribute to numerous diseases.^8^ Competent degradation of extracellular DNA in circulation is of pivotal importance for homeostasis and survival. Wild‐type mice, in which sterile neutrophilia was induced by granulocyte‐colony‐stimulating factor overexpression, survived without any adverse phenotype. In contrast, if DNase 1 and 1L3 were knocked out, neutrophilic mice died within days due to disseminated intravascular coagulation. Even in the absence of fibrin and platelets, DNA sufficed to form occlusive clots in small arteries.^10^

Biomarkers, which allow robust risk stratification and outcome prediction for AS patients undergoing TAVR, would be a valuable tool in routine care. The traditional cardiac markers troponin and BNP were evaluated as predictors for outcome in several reports; while troponin showed differing results, BNP levels proved to predict survival after TAVR.^22^ In our cohort, BNP levels were not significantly connected to survival; however, BNP levels could only be obtained in about half of the patients. Studies on CRP as a predictor for outcome also yielded controversial results.^22^ In the present study, higher CRP was associated with death after 12 months in univariate analysis, but lost significance in the multivariate analysis (Table 2). Galectin 3 and ST2 are further interesting biomarkers to predict outcome in TAVR patients.^23^, ^24^

DNase activity was significantly lower in AS patients compared to the healthy reference control group (Figure 2). Importantly, the groups were not age‐matched (TAVR recipients’ median age 82 years, healthy controls median age 65 years). Although no significant correlation between age and DNase activity was detected in the cohorts, we cannot rule out that age could be one explanatory factor for this distinct difference beneath AS. We found that DNase activity independently predicted outcome after 12 months in TAVR patients (adjusted HR 3.11, *P* = .001). In congruence, patients with low DNase activity levels at baseline displayed higher mean pressure gradients and suffered from pronounced dyspnoea 12 months after TAVR (Figures 4 and 5). Quantification of total DNase activity is a robust and reproducible technique, and as of late, rapid and straight‐forward assays are commercially available. Assays which allow to selectively measure DNase 1 and DNase 1L3 activity are currently underway and could enhance the statistical power of DNase activity as a biomarker to predict outcome after TAVR. Most of the other traditional parameters which influence outcome such as age or creatinine did not reach significance in our model. We speculate that—beyond the strength of DNase activity in the model—the narrow age distribution in the rather homogenous patient cohort (median 82 years, IQR 78‐85 years) led to these results. Moreover, the presence of coronary artery disease was not predictive in our model. All patients received coronary angiography before TAVR, and coronary intervention was performed in case of significant coronary disease. This might explain lack of association within the consecutive 12 months.

Double‐stranded DNA is released into circulation during tissue injury and cell death, but also via NET formation.^14^ In thrombotic or inflammatory disease, dsDNA levels are increased.^25^ However, pronounced high systemic levels might only be observable during acute systemic inflammation; in ST‐elevation myocardial infarction patients, strongly increased dsDNA levels were only found locally at the culprit lesion site.^26^ In the present study, we detected an increase of dsDNA levels at 3 and 7 days after TAVR, most likely reflecting postinterventional cell death and inflammatory boost, but no sustained increase in the following months was observed (Figure 1). We have recently reported that the ratio of DNase activity and dsDNA in plasma has incremental power to predict survival after cardiac arrest.^12^ This was not observed in TAVR patients of the present study.

Inflammatory tissue remodelling and fibrotic calcification are important mechanisms involved in bioprosthetic valve degeneration. The antibody‐mediated response to xenoantigens like alpha‐gal is one trigger of low‐grade inflammatory activation towards the bioprosthetic tissue.^27^ It was shown that genetically engineered porcine organs with alpha‐gal knockout transplanted into baboons survive for several years.^28^ Such hypo‐immunogenic tissue could blunt an antibody‐mediated immune response.^29^ In addition to adaptive immune response, innate immune cells appear to play an important role in bioprosthesis degeneration. Neutrophils are abundant in degenerated porcine bioprostheses.^30^ Bioprosthesis and native valve degeneration might share some pathomechanisms; neutrophil count is connected to aortic valve calcification,^31^ and NETs are associated with the progression and outcome of native aortic valve stenosis.^15^ We found that lower baseline DNase activity correlated with higher baseline CRP levels and with higher mean gradient of the bioprosthetic valve 12 months after TAVR (Figure 2 and Figure 5). Thus, DNase function in circulation is not only interesting as a potential biomarker, but may also allow hypothesis generation on its role in the pathomechanism of bioprosthetic valve degeneration. It could be hypothesized that a proinflammatory milieu inhibits DNase release and subsequently leads to local NET persistence. NETs shift monocytes towards a proinflammatory phenotype^26^ and promote their differentiation into fibrocytes.^17^ Peptidylarginine deiminase 4‐knockout mice, which are unable to form NETs, displayed strongly inhibited organ fibrosis compared to wild‐type littermates. The same inhibition of fibrosis was achieved by the application of DNase.^13^ Locally released NETs might lead to bioprosthetic tissue fibrosis and calcification. The potent prothrombotic capacity of NETs could promote platelet and fibrin adhesion and lead to hypoattenuating leaflet thickening and valve thrombosis. Increased levels of DNase in circulating might prevent these effects.

In conclusion, low baseline DNase activity is connected to increased inflammatory state, pronounced symptoms, higher mean valve gradient and poor survival in TAVR recipients after 12 months. Based on these findings, DNase activity may be further explored as a potential biomarker and its potential involvement in bioprosthetic valve deterioration.

### 4.1 Limitations

The present study is retrospective and observational. The control group is not age‐matched; therefore, residual confounding due to differing median age could influence the results shown in Figure 1 and Figure 2. The power of DNase activity as a biomarker should be reproduced in a prospective TAVR trial. Only all‐cause mortality of TAVR recipients was surveyed, and specific causes of death would enhance the informative value in further studies.

## CONFLICT OF INTEREST

We have nothing to declare.

## AUTHORS CONTRIBUTIONS

AM designed the study, analysed/interpreted data and wrote/revised the manuscript. ASO analysed samples, analysed/interpreted data and wrote/revised the manuscript. TMH analysed/interpreted data and wrote/revised the manuscript. TA analysed samples and wrote/revised the manuscript. NJ analysed samples and wrote/revised the manuscript. NGP analysed samples and wrote/revised the manuscript. MM acquired samples/data and wrote/revised the manuscript. HM acquired samples/data and wrote/revised the manuscript. DF acquired samples/data and wrote/revised the manuscript. UH acquired samples and wrote/revised the manuscript. BW analysed/interpreted data and wrote/revised the manuscript. AL acquired samples and wrote/revised the manuscript. BA acquired samples and wrote/revised the manuscript. EWK acquired samples/data and wrote/revised the manuscript. IML designed the study and wrote/revised the manuscript. ML designed the study, acquired samples, analysed/interpreted data and wrote/revised the manuscript.
